# The Mechanisms Underlying α-Amanitin Resistance in *Drosophila melanogaster*: A Microarray Analysis

**DOI:** 10.1371/journal.pone.0093489

**Published:** 2014-04-02

**Authors:** Chelsea L. Mitchell, Michael C. Saul, Liang Lei, Hairong Wei, Thomas Werner

**Affiliations:** 1 Department of Biological Sciences, Michigan Technological University, Houghton, Michigan, United States of America; 2 Department of Zoology, University of Wisconsin-Madison, Madison, Wisconsin, United States of America; 3 School of Forest Resources and Environmental Science, Michigan Technological University, Houghton, Michigan, United States of America; CNRS, France

## Abstract

The rapid evolution of toxin resistance in animals has important consequences for the ecology of species and our economy. Pesticide resistance in insects has been a subject of intensive study; however, very little is known about how *Drosophila* species became resistant to natural toxins with ecological relevance, such as α-amanitin that is produced in deadly poisonous mushrooms. Here we performed a microarray study to elucidate the genes, chromosomal loci, molecular functions, biological processes, and cellular components that contribute to the α-amanitin resistance phenotype in *Drosophila melanogaster*. We suggest that toxin entry blockage through the cuticle, phase I and II detoxification, sequestration in lipid particles, and proteolytic cleavage of α-amanitin contribute in concert to this quantitative trait. We speculate that the resistance to mushroom toxins in *D. melanogaster* and perhaps in mycophagous *Drosophila* species has evolved as cross-resistance to pesticides, other xenobiotic substances, or environmental stress factors.

## Introduction

How species respond to changes in their environment is a central question in biology. Insects and mammals deploy similar genes and detoxification mechanisms to defend against poisons that are present in their prey or in the environment. These include the avoidance of toxic parts of their diet, the excretion, sequestration, metabolic breakdown of the toxins, and mutations in the target proteins to avoid toxin binding [1]. Some of the most striking natural examples of toxin resistance are snake species that feed on poisonous amphibians [2], caterpillars that sequester plant alkaloids in their bodies to deter predators [1], and toxin-resistant soft-shell clams that store algal toxins in their bodies, causing paralytic shellfish poisoning in people who eat the clams [3]. Apart from these natural examples, the use of pesticides against insects has caused very rapidly evolving toxin resistance responses in many pest species [4]–[8], costing the US billions of dollars per year in crop damage and pesticide production [9].

Out of the vast number of eukaryotic organisms that live on our planet, a few dozen of mycophagous *Drosophila* species are able to breed in a variety of very toxic mushrooms, including the deadly poisonous species *Amanita phalloides* (Death Cap) and *Amanita virosa* (Destroying Angel). Among other toxins, these mushrooms contain α-amanitin as their principal toxin, which inhibits the function of RNA-polymerase II and thus brings all mRNA transcription to a halt [10]. These resistant *Drosophila* species can develop on α-amanitin-containing laboratory food [11], [12], showing that the resistance mechanism is not due to the avoidance of toxic parts of the mushrooms. Furthermore, the RNA-polymerase II of all tested mushroom-feeding *Drosophila* species is as sensitive to α-amanitin as it is in sensitive *Drosophila* species [13], showing that target mutations in the RNA polymerase II complex are not likely to confer resistance to mushroom toxins in mycophagous *Drosophila* species.

The model organism *D. melanogaster* is a non-mycophagous species; i.e., it does not use mushrooms as a natural diet. Thus, *D. melanogaster* should not encounter toxins in nature that are solely produced by mushrooms, such as α-amanitin. However, three Asian *D. melanogaster* strains that were collected in the 1960s in Taiwan (Ama-KTT), India (Ama-MI), and Malaysia (Ama-KLM) were shown to be one order of magnitude more resistant to α-amanitin than the sensitive wild-type strain Oregon-R [14]. In these three Asian strains, the resistance to α-amanitin was mapped to two dominantly acting loci: one situated on the left and one on the right arm of chromosome 3. Eighteen years later, a very similar phenomenon was described in a *D. melanogaster* stock collected in California. This stock showed an increased resistance to α-amanitin and surprisingly, the resistance was mapped to the seemingly same two loci on chromosome 3, as in the three Asian stocks. Even in the Californian stock, both loci acted in a dominant fashion [15]. The Californian study concluded with the identification of two candidate genes that might confer the resistance phenotype: *Multidrug resistance 65* (*Mdr65*) on the left arm and *Protein kinase C98E* (*Pkc98E*) on the right arm of chromosome 3. Because PKC98E can phosphorylate MDR proteins [16] and MDR proteins could potentially lead to the excretion of α-amanitin from cells, the question of how *D. melanogaster* evolved α-amanitin resistance appeared to be answered. Although the proposed scenario is simple and elegant, no conclusive evidence has been brought forward yet that demonstrates that any gene is required or necessary to confer resistance to α-amanitin. Thus, the genes that confer mushroom toxin resistance in *D. melanogaster* (and all mycophagous *Drosophila* species) remain elusive.

In this study, we conducted a whole-genome microarray analysis, using an isochromosome stock for chromosomes 2 and 3 of the original α-amanitin-resistant *D. melanogaster* stock Ama-KTT from Taiwan. We hypothesized that genes involved in the excretion, metabolic inactivation, and/or sequestration of α-amanitin will be identified in our microarray, which can pinpoint to the mechanisms responsible for the α-amanitin resistance phenotype. To our surprise, neither *Mdr* genes nor *Pkc98E* were among the up-regulated candidate genes. Instead, we identified genes of the phase I detoxification gene family *Cyp* (Cytochrome P450), and the phase II *Gst* (Glutathione-S-transferase) and *Ugt* (UDP glucuronosyl transferase) gene families, some of which (*Cyp6a2*, *Cyp12d1-d*, and *Cyp12d1-p*) were several hundred-fold constitutively up-regulated in the α-amanitin-resistant fly stock. In addition, we found evidence for the possible involvement of peptidases, lipid particles, cuticular proteins, the Mayor Royal Jelly Protein homolog Yellow, and Salivary Gland Secretion (Sgs) proteins, which could provide additional protection by cleaving or immobilizing α-amanitin, or by blocking its access to cells. Because *D. melanogaster* does not feed on mushrooms in nature and α-amanitin is solely found in mushrooms, we speculate that the resistance to α-amanitin has evolved as cross-resistance to pesticides or other environmental factors that the flies encountered before they were collected in Asia 45 years ago.

## Results

### Experimental Design

**Figure 1:**
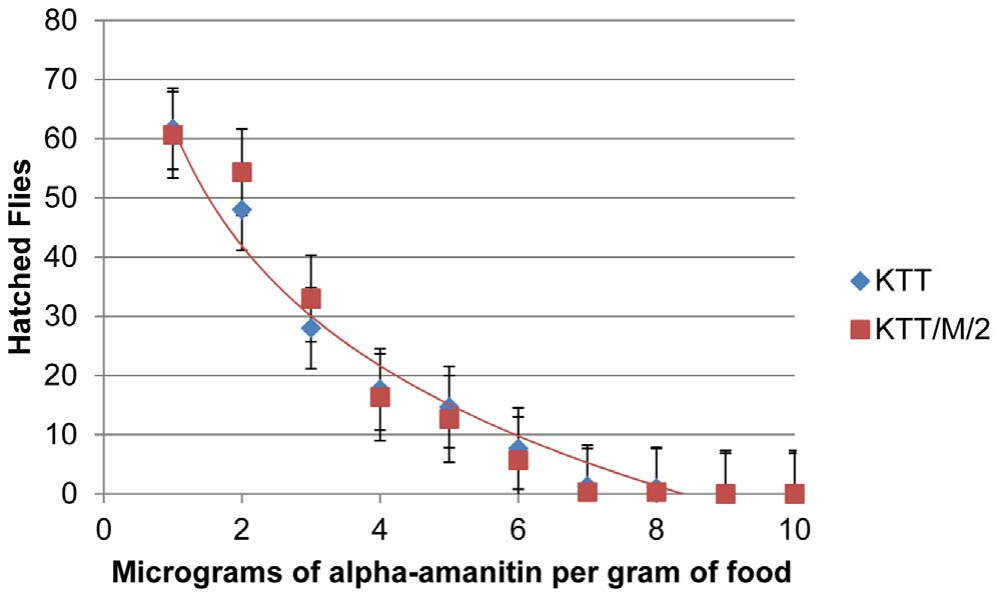
Ama-KTT/M/2 is not less resistant to α-amanitin than Ama-KTT. Ten first-instar larvae were placed on each α-amanitin concentration. The dose response curve shows the percentage of hatching flies. Error bars indicate the s.e.m. of three replicates.

In two independent studies, a total of four *D. melanogaster* stocks from Asia and North America were shown to be resistant to the mushroom toxin α-amanitin [14], [15]. For each of these stocks, QTL mapping data suggested that the resistance was conferred by two dominantly acting loci on chromosome 3. Begun and Whitley identified the genes *Mdr65* and *Pkc98E* as possible candidates, with the notion that the resistance phenotype could be caused by a *cis*-regulatory change in the *Mdr65* gene [15]. In order to identify gene-regulatory changes on a whole-transcriptome scale in α-amanitin-resistant *D. melanogaster* larvae, we performed a microarray study. As starting material, we used the most resistant of the four described α-amanitin-resistant stocks, Ama-KTT [14]. Because the stock could have become heterozygous for the resistance-conferring loci during the past 45 years after being collected in the wild, we created the isochromosome stock Ama-KTT/M/2, which is isogenic for the Ama-KTT chromosomes 2 and 3. Our dose-response data show that the isochromosome stock Ama-KTT/M/2 (LC_50_ = 2.16 μg/g of food) is at least as resistant to α-amanitin as the original Ama-KTT stock (LC_50_ = 1.84 μg/g of food) (Figure 1), indicating that at least the majority of the α-amanitin resistance-conferring genes is located on the major autosomes. The multi-balancer stock that we used for the crosses to create the Ama-KTT/M/2 stock was very sensitive to α-amanitin (LC_50_ = 0.042 μg/g of food, data not shown).

**Figure 2:**
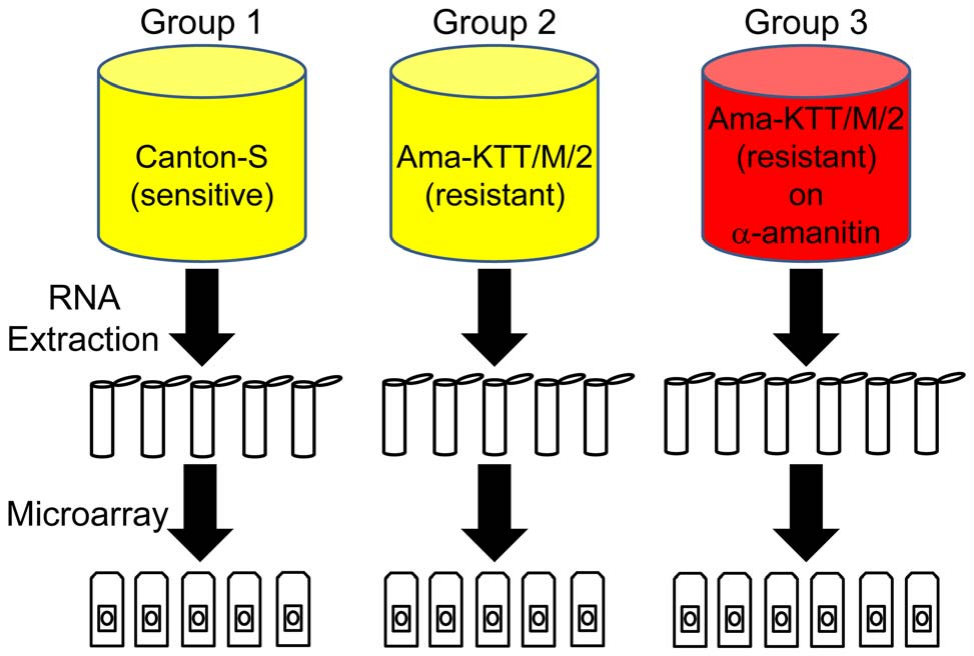
Graphical representation of the groups of larvae used for the microarray and qPCR analysis. Groups 1 and 2 (Canton-S and Ama-KTT/M/2) were not treated with α-amanitin, as symbolized by the yellow color. The larvae of group 3 (Ama-KTT/M/2) were treated with α-amanitin throughout their development, as indicated in red. Groups 1 and 2 were collected in five, and group 3 in six biological replicates (ten larvae in each replicate), as illustrated by the number of tubes and microarray chips.

We performed a whole-transcriptome gene expression microarray analysis to test what genes are differentially expressed in 1) a constitutive manner and 2) in response to α-amanitin. The complete set of differentially expressed genes (DEGs) can be found in Table S1. The isochromosome stock Ama-KTT/M/2 (LC_50_ = 2.16 μg/g of food) was used as the experimental stock and has a 77.1 times higher LC_50_ to α-amanitin than our sensitive control stock Canton-S (LC_50_ = 0.028 μg/g of food, data not shown). We compared three groups with each other: 1) Canton-S larvae on non-toxic food, 2) Ama-KTT/M/2 larvae on non-toxic food, and 3) Ama-KTT/M/2 larvae that were continuously raised from the first to the third instar on α-amanitin-containing food (at 1.5 μg/g of food, a concentration that is slightly below the LC_50_ of Ama-KTT/M/2). Groups 1 and 2 were prepared in 5, and group 3 in six biological replicates, each replicate consisting of ten larvae (Figure 2). We compared the gene expression profiles of fully-grown third-instar larvae that have not started wandering yet. For the data analysis, we focused on well-annotated genes that showed expression changes of at least 2-fold with a corrected p-value of less than 0.05. With the exception of the genome enrichment analysis and the gene *CG10226*, which is a putative *Mdr* gene, we generally excluded genes from our analysis that solely have a CG or CR gene annotation number.

### Genes Encoding Cytochrome P450s, GSTs, and UGTs Are Differentially Expressed in Ama-KTT/M/2

Assuming that gene-regulatory changes underlie α-amanitin resistance in the Ama-KTT/M/2 isochromosome stock, we expected to identify constitutive gene-expression changes in Ama-KTT/M/2 on non-toxic food, as compared to the sensitive control stock Canton-S on non-toxic food (group 2 versus group 1). We used the Plier normalization/summarization and the DEG methods to analyze our single gene microarray data. As a result, we identified 234 genes that were at least 2-fold significantly constitutively up-regulated in Ama-KTT/M/2 (Table S2). Out of these 234 genes, 20 (8.5%) are *Cyp*, *Gst*, and *Ugt* genes, which are all situated on chromosomes 2 and 3 (Table 1). The three most highly up-regulated genes of this group were *Cyp6a2*, *Cyp12d1-d*, and *Cyp12d1-p*, which were between more than 300- to 197.3-fold constitutively up-regulated in the resistant stock. These three genes are expressed in the larval midgut and Malpighian tubules, which are potential detoxification organs [17]. Interestingly, *Cyp6a2* expression profiles are correlated with insecticide resistance [18]–[24], while CYP6A2 metabolizes insecticides in enzyme assays [19], [25]. *Cyp12d1* is also associated with insecticide resistance [22], [23], [26]–[31] and stress response [29]–[31]. Overexpression of *Cyp12d1* increases insecticide resistance [32], and CYP12D1 from the house fly metabolizes insecticides [33]. The remaining 17 detoxification genes identified in our microarray study were 38.9 - 2.1-fold up-regulated and are presented next in the order from the highest to lowest constitutive up-regulation in Ama-KTT/M/2: *Ugt36Bb*, *Ugt86Dd*, *GstD5*, *GstE1*, *GstE6*, *GstE5*, *Ugt36Bc*, *Cyp6a20*, *Ugt37c1*, *Ugt36Ba*, *Cyp4c3*, *Ugt37b1*,*Cyp6w1*, *Cyp305a1*, *Cyp49a1*, *GstD8*, and *GstE9*. Some of these genes are associated various phenotypes: *Ugt86Dd* and *Cyp6w1* (inducibility by the xenobiotic phenobarbital) [23], *GstD5* and *GstE1* (stress responses) [34], [35], *GstE5* (insecticide resistance) [36], *Cyp6a20* (aggressive behavior) [37]–[39], and *Cyp305a1* (ecdysteroid synthesis and lipid storage regulation) [40].

**Table 1:** Single gene analysis for Ama-KTT/M/2 versus Canton-S on no toxin (group 2 versus 1).

Gene Symbol	Chromosome	Fold Change	p-Value	FlyBase ID	Probe ID
*Cyp6a2*	2R	**>300**	0	FBgn0000473	1626401_at
*Cyp12d1-d*	2R	**280.1**	0	FBgn0053503	1639069_at
*Cyp12d1-d///Cyp12d1-p*	2R	**197.3**	0	FBgn0050489///FBgn0053503	1633401_s_at
*Ugt36Bb*	2L	**38.9**	0.00345	FBgn0040261	1625402_at
*yellow*	X	**14.7**	0.04477	FBgn0004034	1633285_at
*Ugt86Dd*	3R	**12.5**	0	FBgn0040256	1641481_at
*GstD5*	3R	**10.1**	0	FBgn0010041	1634152_at
*GstE1*	2R	**9.8**	0	FBgn0034335	1623256_at
*GstE6*	2R	**8.8**	0	FBgn0063494	1625744_at
*GstE5*	2R	**7.1**	0	FBgn0063495	1624732_at
*Ugt36Bc*	2L	**7.0**	0	FBgn0040260	1641191_s_at
*Cyp6a20*	2R	**4.7**	0.02639	FBgn0033980	1632021_at
*Ugt37c1*	2R	**2.9**	0.00200	FBgn0026754	1639299_at
*Ugt36Ba*	2L	**2.9**	0.00348	FBgn0040262	1629836_at
*Cyp4c3*	3R	**2.8**	0.02333	FBgn0015032	1636716_at
*Ugt37b1*	2L	**2.6**	0.00352	FBgn0026755	1640109_at
*Cyp6w1*	2R	**2.4**	0	FBgn0033065	1634143_at
*Cyp305a1*	3L	**2.3**	0.01461	FBgn0036910	1628584_at
*Cyp49a1*	2R	**2.1**	0.03070	FBgn0033524	1639901_a_at
*GstD8*	3R	**2.1**	0.03157	FBgn0010044	1634554_at
*GstE9*	2R	**2.1**	0	FBgn0063491	1628657_at
*Cyp12a4*	3R	**−2.1**	0	FBgn0038681	1632114_at
*Cyp304a1*	3R	**−2.1**	0.02226	FBgn0038095	1632451_at
*Cyp313a2*	3R	**−2.3**	0	FBgn0038006	1623727_at
*Cyp12e1*	3R	**−2.6**	0	FBgn0037817	1626022_at
*Cyp6t1*	X	**−2.7**	0.04340	FBgn0031182	1626689_at
*Cyp4ac2*	2L	**−2.7**	0	FBgn0031694	1623866_at
*Cyp4s3*	X	**−3.2**	0.00126	FBgn0030615	1636688_at
*Ugt86Dj*	3R	**−3.4**	0.02615	FBgn0040250	1634029_at
*Cyp4d2*	X	**−3.6**	0	FBgn0011576	1636793_at
*Cyp6a23*	2R	**−5.4**	0	FBgn0033978	1624101_at
*Cyp4ac3*	2L	**−6.1**	0	FBgn0031695	1638739_at
*Cyp4p2*	2R	**−6.5**	0.00137	FBgn0033395	1640566_at
*Cyp28d1*	2L	**−6.9**	0	FBgn0031689	1633639_at
*Cyp4d8*	3L	**−7.6**	0	FBgn0015033	1626198_at
*Cyp6a17*	2R	**−186.8**	0	FBgn0015714	1628052_at
*Mdr50*	2R	1.6	0.00648	FBgn0010241	1638775_at
*CG10226*	3L	1.4	0	FBgn0035695	1632500_at
*Mdr65*	3L	1.2	0	FBgn0004513	1631925_at
*Mdr49*	2R	1.1	0	FBgn0004512	1628659_at
*Pkc98E*	3R	−1.1	0.13512	FBgn0003093	1631059_at
*cnc*	3R	−1.2	0.00131	FBgn0000338	1633379_s_at
*Hr96*	3R	−1.6	0.00142	FBgn0015240	1639398_at

Type I and II detoxification, *Mdr*, and transcription factor genes with possible functions in detoxification processes are shown. The at least 2-fold differentially expressed genes are sorted by positive and negative fold-changes, followed by the genes that are not significantly differentially expressed. All p-values are corrected. The chromosomes, FlyBaseID, and probe ID numbers are presented.

We were curious to see if the constitutive up-regulation of detoxification genes is a specific characteristic for the α-amanitin-resistant stock Ama-KTT/M/2 or if there are other detoxification genes that show higher expression levels in Canton-S, as compared to Ama-KTT/M/2. Surprisingly, 15 *Cyp* and *Ugt* genes were between 2.1 and 186.8-fold lower expressed in the resistant stock Ama-KTT/M/2 than in Canton-S. From the lowest to highest expression difference, these genes are: *Cyp12a4*, *Cyp304a1*, *Cyp313a2*, *Cyp12e1*, *Cyp6t1*, *Cyp4ac2*, *Cyp4s3*, *Ugt86Dj*, *Cyp4d2*, *Cyp6a23*, *Cyp4ac3*, *Cyp4p2*, *Cyp28d1*, *Cyp4d8*, and *Cyp6a17* (Table 1). Correlative or functional data exists for *Cyp12a4* and *Cyp4p2* (insecticide resistance) [21], [41], *Cyp304a1* and *Cyp4d2* (methanol resistance) [42], and *Cyp6a17* (thermosensory behavior) [43].

### Genes Encoding Cytochrome P450s and GSTs Are Inducible by α-Amanitin

Our next question was what genes are inducible by α-amanitin in the resistant Ama-KTT/M/2 stock as compared to Ama-KTT/M/2 on non-toxic food (group 3 versus group 2). We found that 143 genes were significantly inducible by α-amanitin (Table S3), eleven of which (7.7%) belong to the *Cyp* and *Gst* gene families (Table 2). *Cyp316a1* was the strongest inducible *Cyp* gene (11.8-fold) in the resistant stock Ama-KTT/M/2. However, when we compared resistant Ama-KTT/M/2 on toxic food to sensitive Canton S without toxin (group 3 versus group 1), *Cyp316a1* was only 1.9-fold (p = 0.0941, Table S1) more expressed in Ama-KTT/M/2 on toxic food, making the 11.8-fold induction within the Ama-KTT/M/2 stock less convincing. The remaining ten *Cyp* and *Gst* that were up-regulated by α-amanitin in the resistant stock were induced between 7.2- and 2.0-fold and are listed in the order from highest to lowest induction: *Cyp6d2*, *Cyp4d8*, *Cyp28d1*, *Cyp6t1*, *GstD3*, *GstD6*, *Cyp4d2*, *GstD9*, *GstD10*, and *Cyp4d14*. Four of these genes, *Cyp4d8*, *Cyp28d1*, *Cyp4d2*, and *Cyp4d14*, are expressed in the larval midgut and/or Malpighian tubules, suggesting that they could play a role in the detoxification of xenobiotic compounds [17]. Some genes are associated with various phenotypes: *Cyp6d2* (camptothecin resistance) [44], *GstD6* (oxidative stress response) [45], and *Cyp4d2* (methanol resistance) [42]. Notably, both *Cyp316a1* and *Cyp4d8* are situated at cytological position 66A2, which is relatively close to region 65A10 to which α-amanitin resistance was QTL-mapped in four independent *D. melanogaster* stocks in the past [14], [15]. We next asked what *Cyp*, *Gst*, and *Ugt* genes were down-regulated in response to α-amanitin in the resistant stock. As a result, nine genes were 2.1- to 3.8-fold down-regulated in response to α-amanitin, which are presented in the order from lowest to highest down-regulation: *Ugt37b1*, *Cyp4c3*, *Cyp28d2*, *Ugt86Dd*, *Cyp6a23*, *Cyp9b2*, *Ugt37c1*, and *Cyp28a5* (Table 2). Out of these, *Ugt86Dd* is inducible by the xenobiotic phenobarbital [23] and *Cyp28a5* by methanol [42]. Some of the most strongly α-amanitin-inducible genes (>300-fold) were the salivary gland secretion genes *Sgs1*, *Sgs3*, *Sgs5*, *Sgs7*, and *Sgs8* (Table S3). We will speculate about their role later.

**Table 2:** Single gene analysis for Ama-KTT/M/2 on α-amanitin versus Ama-KTT/M/2 on no toxin (group 3 versus 2).

Gene Symbol	Chromosome	Fold Change	p-Value	FlyBase ID	Probe ID
*Cyp316a1*	3L	**11.8**	0.01038	FBgn0035790	1634540_at
*Cyp6d2*	2R	**7.2**	0	FBgn0034756	1635593_at
*Cyp4d8*	3L	**7.1**	0	FBgn0015033	1626198_at
*Cyp28d1*	2L	**6.5**	0	FBgn0031689	1633639_at
*Cyp6t1*	X	**4.0**	0.00705	FBgn0031182	1626689_at
*GstD3*	3R	**3.4**	0	FBgn0010039	1635701_at
*GstD6*	3R	**3.2**	0	FBgn0010042	1626136_at
*Cyp4d2*	X	**2.6**	0	FBgn0011576	1636793_at
*GstD9*	3R	**2.2**	0	FBgn0038020	1636174_at
*GstD10*	3R	**2.1**	0	FBgn0042206	1627890_at
*Cyp4d14*	X	**2.0**	0	FBgn0023541	1627180_at
*Ugt37b1*	2L	**−2.1**	0.00217	FBgn0026755	1640109_at
*Cyp4c3*	3R	**−2.2**	0.01823	FBgn0015032	1636716_at
*Cyp28d2*	2L	**−2.2**	0.03701	FBgn0031688	1624911_at
*Ugt86Dd*	3R	**−2.8**	0	FBgn0040256	1641481_at
*Cyp6a23*	2R	**−2.8**	0.03644	FBgn0033978	1624101_at
*Cyp9b2*	2R	**−3.0**	0	FBgn0015039	1635008_at
*Ugt37c1*	2R	**−3.2**	0.00116	FBgn0026754	1639299_at
*Mdr50*	2R	**−3.3**	0	FBgn0010241	1638775_at
*Cyp28a5*	2L	**−3.8**	0	FBgn0028940	1629009_at
*cnc*	3R	1.2	0	FBgn0000338	1633379_s_at
*Pkc98E*	3R	1.2	0.05111	FBgn0003093	1631059_at
*Mdr49*	2R	1.1	0.17186	FBgn0004512	1628659_at
*CG10226*	3L	−1.2	0	FBgn0035695	1632500_at
*Mdr65*	3L	−1.2	0	FBgn0004513	1631925_at
*Hr96*	3R	−1.2	0.08263	FBgn0015240	1639398_at

Type I and II detoxification, *Mdr*, and transcription factor genes with possible functions in detoxification processes are shown, sorted by positive and negative fold-changes. The at least 2-fold differentially expressed genes are sorted by positive and negative fold-changes, followed by the genes that are not significantly differentially expressed. All p-values are corrected. The chromosomes, FlyBaseID, and probe ID numbers are presented.

### *Mdr* Genes Are Neither Constitutively Up-Regulated nor Inducible in Ama-KTT/M/2

In 1982, QTL mapping data suggested that two loci on chromosome 3 of the Asian Ama-KTT, Ama-MI and Ama-KLM stocks confer resistance to α-amanitin in a dominant fashion [14]. Eighteen years later, a Californian *D. melanogaster* stock showed α-amanitin resistance that was QTL-mapped to virtually the same two loci on chromosome 3 [15]. It was concluded that *Mdr65* and *Pkc98E* were possible candidate genes for causing the resistance. Furthermore, sequence comparisons between the most and the least resistant Californian stocks pointed out differences in the non-coding regions, but not in the coding regions of *Mdr65*. Thus, if *Mdr65* would confer resistance, the prediction was that a *cis*-regulatory change in the *Mdr65* gene is responsible for the resistance α-amanitin. We thus asked the question if *Pkc98E*, *Mdr65* or any other *Mdr* genes (*CG10226*, *Mdr49*, and *Mdr50*) were either constitutively up-regulated or inducible by α-amanitin in the Ama-KTT/M/2 stock. Comparing group 2 with group 1, *Mdr65* showed a statistically significant but very low (1.2-fold) constitutive up-regulation in Ama-KTT/M/2 (Table 1), while *Mdr65* was 1.2-fold down-regulated in response to α-amanitin when group 3 was compared to group 2 (Table 2). *CG10226*, a predicted *Mdr* gene that directly flanks the *Mdr65* gene on the left arm of chromosome 3, showed a statistically significant 1.4-fold constitutive up-regulation in the resistant Ama-KTT/M/2 stock as compared to Canton-S (Table 1), while this gene was 1.2-fold down-regulated in response to α-amanitin (Table 2). The remaining two *Mdr* genes of *D. melanogaster*, *Mdr49* and *Mdr50*, are both situated on the right arm of chromosome 2. *Mdr49* showed a mere 1.1-fold constitutive up-regulation in Ama-KTT/M/2 (Table 1), and it is 1.1-fold inducible by α-amanitin (the latter value is statistically insignificant) (Table 2). The observed 1.6-fold constitutive induction of the *Mdr50* gene was statistically significant (Table 1), and the same gene was significantly 3.3-fold down-regulated in response to α-amanitin (Table 2). Furthermore, *Pkc98* is 1.1 times lower expressed in Ama-KTT/M/2 as compared to Canton-S on no toxin (Table 1), while this gene is 1.2-fold inducible by α-amanitin (both values statistically insignificant) (Table 2). In summary, our data show that *Mdr* genes and *Pkc98E* were far less than 2-fold (if at all) up-regulated, neither constitutively nor in response to α-amanitin. *Mdr* genes are thus not likely to confer the α-amanitin resistance, at least not by increasing *Mdr* gene expression.

We also specifically analyzed the regulation of two transcription factor genes that are known to play a role in regulating responses to xenobiotic factors. *Hr96* encodes a nuclear receptor that is involved in xenobiotic responses in *D. melanogaster* [46]. Our data in Table 1 show that *Hr96* is 1.6-fold constitutively higher expressed in Canton-S (group 1) than in Ama-KTT/M/2 on no toxin (group 2). In response to α-amanitin, the *Hr96* gene was 1.2-fold (statistically insignificant) down-regulated in Ama-KTT/M/2 (group 3 versus group 2, Table 2). The other gene of interest was the leucine zipper transcription factor *cnc*, which is known to activate oxidative stress and detoxification responses in *D. melanogaster* [47], [48]. In our microarray, the *cnc* gene is 1.2-fold constitutively higher expressed in Canton-S (group 1) than Ama-KTT/M/2 (group 2, Table 1), while α-amanitin treatment caused a1.2-fold induction in the Ama-KTT/M/2 stock (group 3 versus group 2, Table 2), thus bringing *cnc* gene expression to the same level that was observed in Canton-S without toxin. These results, at least at the transcriptional level, do not suggest the involvement of both transcription factors in the resistance to α-amanitin.

### Genome Enrichment Analysis Confirms 30-Year-Old QTL Mapping Data

**Figure 3:**
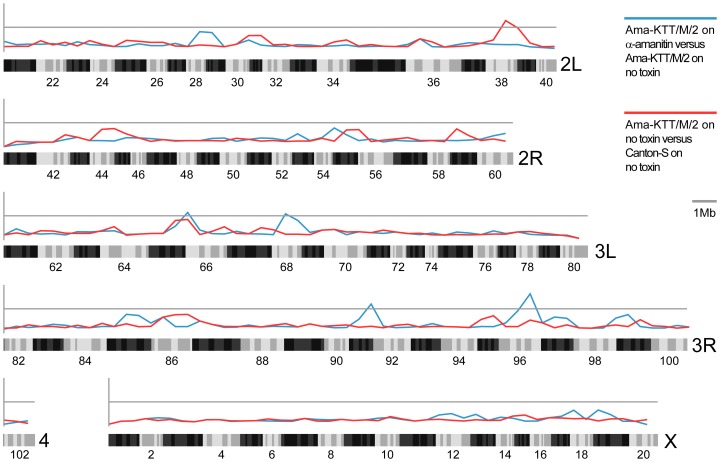
Genome enrichment analysis for genomic correlates. Genomic correlates are likely disrupted in Ama-KTT/M/2 versus Canton S (red) and Ama-KTT/M/2 on α-amanitin versus Ama-KTT on non-toxic food (blue). Colored lines above the gray line indicate significant enrichment of a genomic correlate. Of the five genomic correlates rising above the cutoff value, two genomic correlates are similar to those found in previous linkage studies on the Ama-KTT stock.

In order to identify the regulatory pathway components that lead to the α-amanitin resistance phenotype, we performed a genome enrichment analysis to look for clusters of significantly differentially expressed genes along the four chromosomes. In accordance with the two previous studies that mapped α-amanitin resistance to the polytene bands 95 and 98 on chromosome 3, we found signatures for both constitutive (group 2 versus group 1) and α-amanitin-inducible (group 3 versus group 2) clusters of differentially expressed genes. The only constitutively differentially expressed gene cluster is situated at cytological band 38B on the left arm of chromosome 2, which contains the genes *CG10659*, *Taf13*, *CG17570*, *phr6-4*, *dia*, and *CG31674* at the peak of differential expression (Figure 3 and Table S4). However, their predicted and experimentally proven functions do not explain how α-amanitin resistance is genetically controlled. The remaining four clusters of differentially expressed genes responded to α-amanitin in the larval food. The most interesting induced gene cluster is situated at cytological band 66A, which is close to *Mdr65*-containing region 65A10 on the left arm of chromosome 3, to which α-amanitin resistance was previously mapped [14], [15]. The genes at the peak of differential expression are *mp*, *Hsc70-4*, *pst*, *CG8562*, *Cyp316a1*, *Cyp4d8*, *CG33276*, and *RNaseX25* (Figure 3 and Table S4). Interestingly, two predicted Cytochrome P450 genes with unknown functions, *Cyp316a1* and *Cyp4d8*, were 11.8- and 7.1-fold inducible by α-amanitin (see also Table 2). We further identified differentially expressed gene clusters at cytological bands 68A (left arm of chromosome 3), 92A, and 96D (right arm of chromosome 3). Most of these genes are poorly annotated and none of the genes were linked to any known toxin response (Figure 3 and Table S4). It is worth noting that the transcription factor gene *Hr96* is close to the previously identified *Pkc98* locus, to which α-amanitin resistance was mapped [14], [15]. Although our single gene analysis did not show significant up-regulation of the *Hr96* gene, it is nevertheless possible that Hr96 contributes to the resistance on a post-transcriptional level.

### Gene Ontology Enrichment Analysis Suggests Additional α-Amanitin Resistance Mechanisms

**Figure 4:**
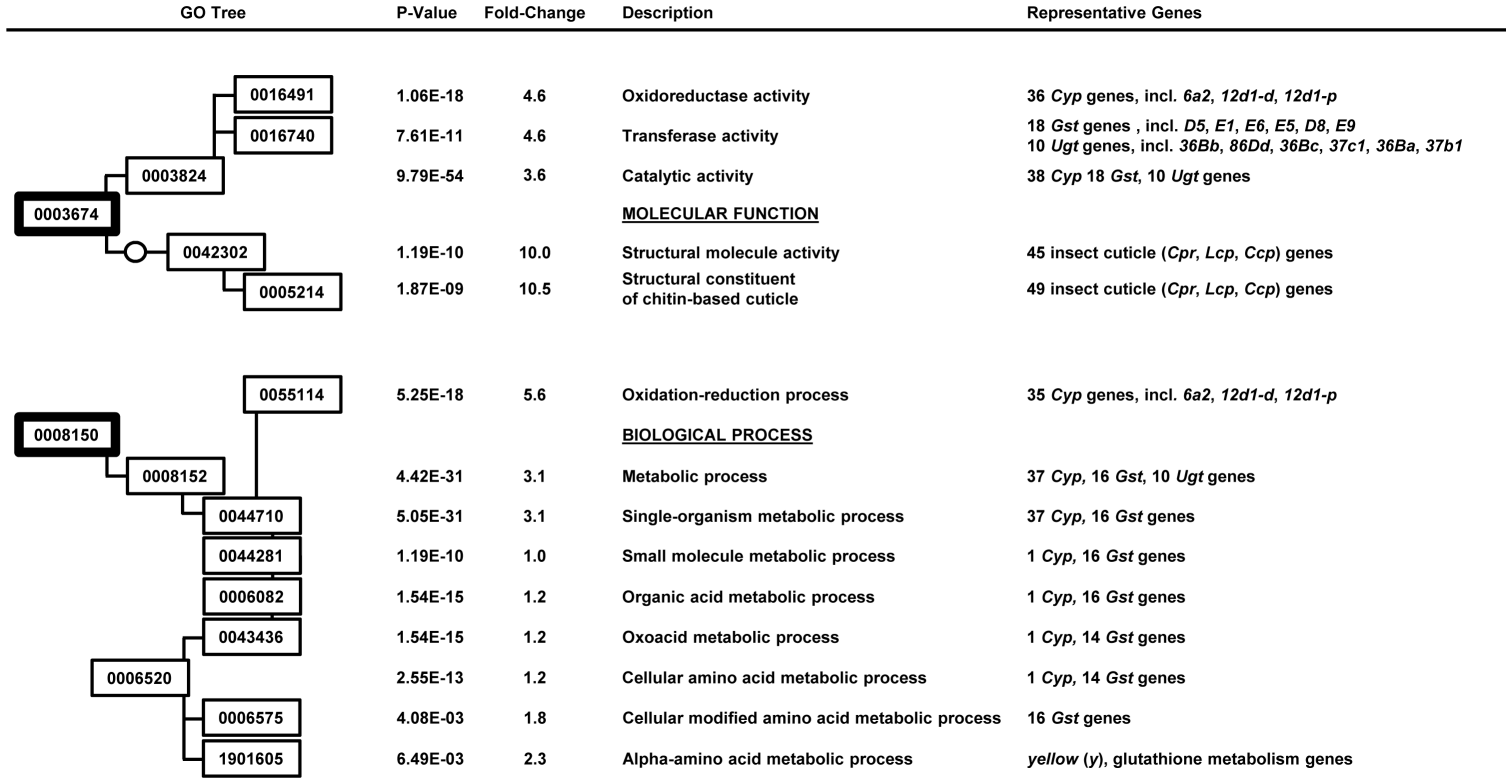
Gene ontology enrichment analysis for Ama-KTT/M/2 versus Canton-S on no toxin (group 2 versus 1). The GO trees for the molecular function and biological process are shown on the left-hand side with the numbers for each term. The corrected p-values, average fold-changes for all genes in each term, term names, and selected genes of each GO term are shown on the right-hand side of each term number.

In order to explore if multiple mechanisms confer the resistance phenotype to α-amanitin in the Ama-KTT/M/2 stock, we performed a gene ontology enrichment analysis. First, we compared the constitutive gene expression differences between Ama-KTT/M/2 and Canton-S on non-toxic food (group 2 versus group 1). As a result, we identified three molecular functions that could be relevant for the α-amanitin resistance phenotype in Ama-KTT/M/2 (Figure 4): 1) ‘Oxidoreductase activity’ genes (GO 0016491) were on average 4.6-fold higher expressed (p = 1.06E-18) in Ama-KTT/M/2. This result confirms the single gene analysis results (Table 1), which indicated that the three highest constitutively expressed *Cyp* genes (*Cyp6a2*, *Cyp12d1-d*, and *Cyp12d1-p* might be important for the resistance to α-amanitin. 2) ‘Transferase activity’ genes (GO 0016740) were on average 4.6-fold higher expressed in Ama-KTT/M/2 (p = 7.61E-11), confirming our single gene analysis for the *Gst and Ugt* genes (Table 1). 3) ‘Structural constituents of chitin-based cuticle’ genes (GO 0005214) were on average 10.5-fold (p = 1.87E-18) higher expressed in Ama-KTT/M/2, including 45 insect cuticle genes of the *Cpr*, *Lcp*, and *Ccp* gene families, which belong to the top 190 constitutively up-regulated genes in Ama-KTT/M/2 (Table S2). It is possible that cuticular proteins provide a protective layer against α-amanitin in organs that are covered by a cuticle, such as the epidermis and the gut. For example in honey bees, ‘structural constituents of chitin-based cuticle’ genes have been suggested to protect venom gland cells from toxins that are stored in the gland [49]. It is interesting to note that like α-amanitin, the bee venom ingredient Mast Cell Degranulating (MCD) Peptide is a bicyclic peptide. Structural constituents of the chitin-based cuticle could perhaps bind to bicyclic peptides and prevent them from entering cells. Furthermore, we identified two significant biological processes in this comparison (group 2 versus group 1) (Figure 4). 1) ‘Oxidation-reduction process’ genes (GO 0055114) were on average 5.6-fold higher expressed in Ama-KTT/M/2 (p = 5.25E-18), confirming the possible role of *Cyp* genes in α-amanitin detoxification. 2) The ‘cellular amino acid metabolic process’ genes (GO 0006520) showed a 1.2-fold higher expression average in Ama-KTT/M/2 (p = 2.55E-13) and was divided into two sub-processes. 2a) The ‘cellular modified amino acid process’ (GO 0006575) contained 16 *Gst* genes, which were on average 1.8-fold higher expressed in Ama-KTT/M/2 (p = 4.08E-03), suggesting that GST enzymes might help detoxifying α-amanitin via the phase II detoxification process. 2b) ‘Alpha-amino acid metabolic process’ genes (GO 1901605), such as glutathione metabolism genes, were on average 2.3-fold constitutively up-regulated in Ama-KTT/M/2 (p = 6.49E-03). Some of these genes might provide the substrate glutathione for the GST enzymes. Interestingly, *yellow* (*y*), a well-known pigmentation gene in *Drosophila*, was among the genes of this gene ontology term (14.7-fold up-regulated, p = 0.0448, Table S2). *yellow* is closely related to *Major Royal Jelly Protein* (*MRJP*) genes in honey bees, which were previously suggested to protect the venom gland cells from the bee venom [49]. It is thus possible that *yellow* plays a role in keeping α-amanitin outside of tissues or perhaps even modifying it so that it becomes less toxic.

**Figure 5:**
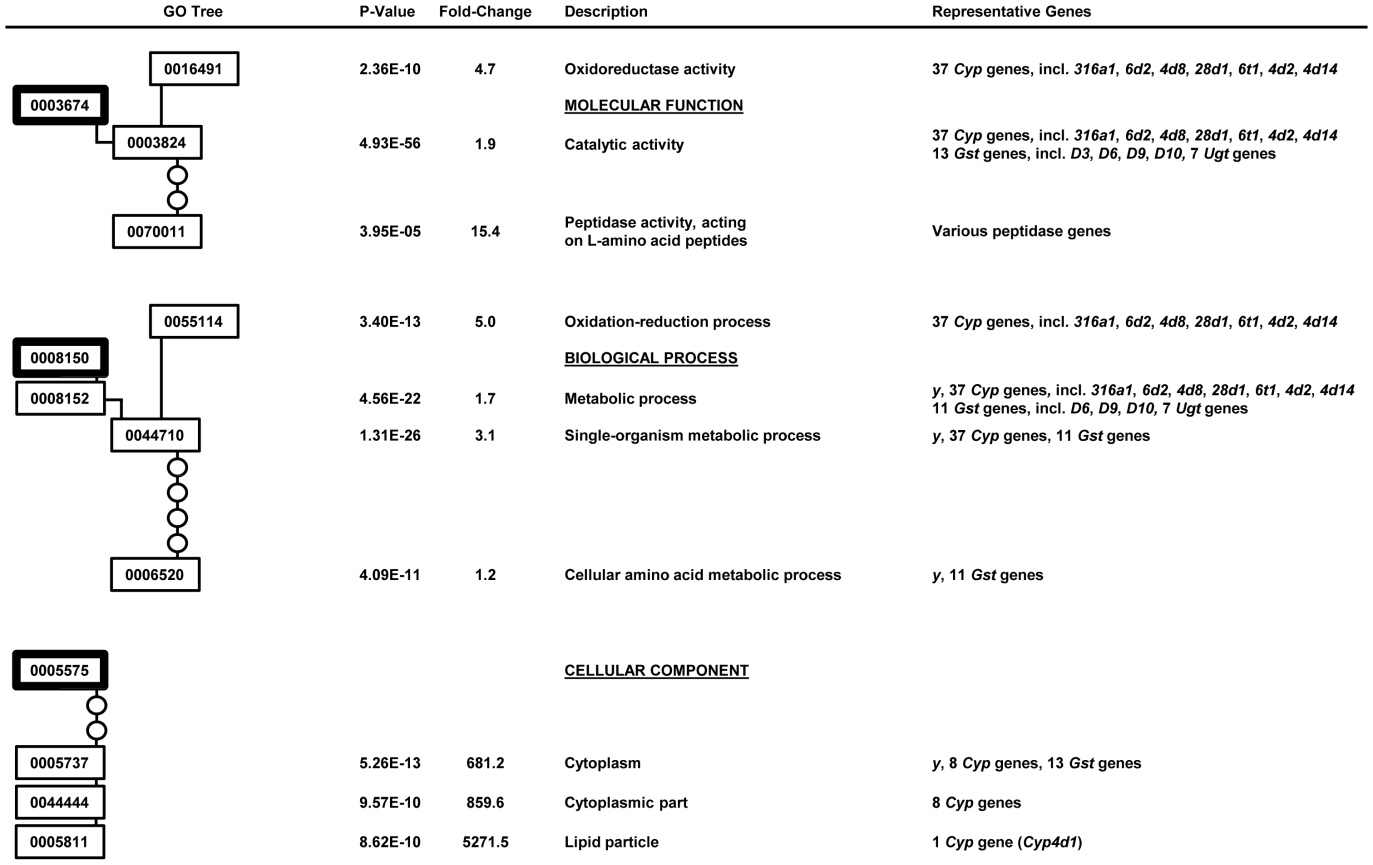
Gene ontology enrichment analysis for Ama-KTT/M/2 on α-amanitin versus Ama-KTT/M/2 on no toxin (group 3 versus 2). The GO trees for the molecular function, biological process, and cellular component are shown on the left-hand side with the numbers for each term. The corrected p-values, average fold-changes for all genes in each term, term names, and selected genes of each GO term are shown on the right-hand side of each term number.

Next, we aimed to identify the gene ontologies that respond to α-amanitin in the resistant stock Ama-KTT/M/2. We thus compared Ama-KTT/M/2 on α-amanitin-containing food to Ama-KTT/M/2 on non-toxic food (group 3 versus group 2). As a result, we identified genes with two molecular functions that are significantly induced by α-amanitin (Figure 5). 1) The ‘oxidoreductase activity’ genes (GO 0016491) are on average 4.7-fold induced (p = 2.36E-10) by the toxin, again suggesting that a phase I detoxification process mediated by Cytochrome P450s is involved in conferring α-amanitin resistance. Among the 37 *Cyp* genes of this gene ontology term, we found seven genes that we already identified in our single gene analysis (Table 2): *Cyp316a1*, *Cyp6d2*, *Cyp4d8*, *Cyp28d1*, *Cyp6t1*, *Cyp4d2*, and *Cyp4d14*. 2) ‘Peptidase activity, acting on L-amino acid peptides’ genes (GO 0070011) were on average 15.4-fold induced (p = 3.95E-05). Because α-amanitin is a peptide, peptidases are good candidates to cleave it. To date, however, no specific enzyme is known that can inactivate α-amanitin by cleaving this bicyclic octapeptide. Besides molecular functions, we further identified two biological processes that were of interest. 1) The ‘oxidation-reduction process’ genes (GO 0055114) were on average 5.0-fold induced (p = 3.40E-13), again confirming that *Cyp* genes could play a role in detoxifying α-amanitin. 2) We identified the ‘cellular amino acid metabolic process’ (GO 0006520) with an average up-regulation of 1.2-fold (p = 4.09E-11) in response to α-amanitin. The most interesting genes in this gene ontology group are 11 *Gst* genes and the *yellow* gene, again showing that the phase II detoxification process is inducible by α-amanitin and that *yellow* could play a protective role. Our gene ontology enrichment analysis further identified cellular components that respond to α-amanitin exposure (Figure 5). 1) ‘Cytoplasm’ genes (GO 0005737) were on average 681.2-fold induced (p = 5.26E-13), some of which are *yellow*, eight *Cyp* genes, and 13 *Gst* genes. The eight *Cyp* genes belong to the gene ontology term ‘cytoplasmic part’ (GO 0044444), which is on average 859.6-fold induced (p = 9.57E-10). Unexpectedly, the most highly induced gene ontology term for the cellular component was the ‘lipid particle’ with an average gene induction of 5,271.5-fold (p = 8.62E-10). Lipid particles are subcellular structures that play roles in detoxification processes and the innate immune system. In insects, lipid particles form coagulation products, thereby protecting cells from pathogens and toxic products of the phenol oxidase cascade [50]. In yeast cells, lipid particles detoxify excessive amounts of lipophilic substances [51]. Even in humans, liposomes are used for detoxifying patients with overdoses of drugs, such as heroin, opioids, and cocaine [52]. The fact that the Ama-KTT/M/2 stock responds to α-amanitin with a several thousand-fold induction of lipid particle genes suggests that cytoplasmic lipid particles contribute to the resistance to α-amanitin in the Ama-KTT/M/2 stock.

### The Domain Enrichment Analysis Verifies the Gene Ontology Enrichment Analysis

Because many proteins have more than one functional domain and the gene ontology enrichment analysis cannot reveal what domain of a protein is important for the resistance to α-amanitin, we further performed a domain enrichment analysis with our microarray data. As shown in Table 3, when comparing Ama-KTT/M/2 with Canton-S (group 2 versus group 1) on non-toxic food, the following protein domains were identified as significantly enriched: Cytochrome P450 (p = 4.72E-11), UDP-glucuronosyl/UDP-glucosyltransferase (p = 1.26E-10), Cytochrome P450, conserved site (p = 5.93E-10), insect cuticle protein (p = 1.55E-09), Cytochrome P450, E-class, group I (p = 6.05E-09), Glutathione S-transferase, C-terminal (p = 6.39E-06), Glutathione S-transferase, C-terminal-like (p = 1.02E-05), Glutathione S-transferase/chloride channel, C-terminal (p = 1.28E-05), and Glutathione S-transferase, N-terminal (p = 4.77E-05). Thus, the domain enrichment analysis confirms the possible importance of phase I and II detoxification reactions in conferring α-amanitin resistance. When comparing Ama-KTT/M/2 on α-amanitin-containing food to Ama-KTT/M/2 on no toxin (group 3 versus group 2, Table 4), we identified the following significantly enriched protein domains: major royal jelly (p = 0), pupal cuticle protein C1 (p = 0), Cytochrome P450 (p = 1.20E-12), Cytochrome P450, conserved site (p = 2.90E-12), insect cuticle protein (p = 1.91E-11), chitin binding domain (p = 3.38E-11), Cytochrome P450, E-class, group I (p = 1.77E-10), peptidase M17, leucyl aminopeptidase, N-terminal (p = 4.38E-06), UDP-glucuronosyl/UDP-glucosyltransferase (p = 4.93E-06), leucine aminopeptidase/peptidase B (p = 8.48E-06), and peptidase M17, leucyl aminopeptidase, C-terminal (p = 8.49E-06). These results confirm the results from the gene ontology enrichment analysis, suggesting that Cytochrome P450s and transferases can detoxify α-amanitin via the phase I and II detoxification pathways. Furthermore, peptidases might cleave α-amanitin, and Royal Jelly Protein domain-containing proteins might protect tissues from α-amanitin, similar to the situation in the honey bee venom gland [49].

**Table 3:** Domain enrichment analysis for Ama-KTT/M/2 versus Canton-S on no toxin (group 2 versus 1).

Domain	DEGs w/Domain	DEGs	Genes w/Domain	Genes	p-Value
Cytochrome P450	48	2609	91	11890	4.72E-11
UDP-glucuronosyl/UDP-glucosyltransferase	25	2609	35	11890	1.26E-10
Cytochrome P450, conserved site	43	2609	82	11890	5.93E-10
Insect cuticle protein	51	2609	107	11890	1.55E-09
Cytochrome P450, E-class, group I	41	2609	81	11890	6.05E-09
Glutathione S-transferase, C-terminal	21	2609	39	11890	6.39E-06
Glutathione S-transferase, C-terminal-like	25	2609	51	11890	1.02E-05
Glutathione S-transferase/chloride channel, C-term.	22	2609	43	11890	1.28E-05
Glutathione S-transferase, N-terminal	20	2609	40	11890	4.77E-05

This table shows the selected and significantly enriched domains without toxin treatment. “DEGs w/domain” are the differentially expressed genes that have a particular domain. “DEGs” is the number of all differentially expressed genes in this comparison. “Genes w/domain” is the total number of genes with a particular domain in the genome. “Genes” is the total number of genes in the genome. All p-values are corrected.

**Table 4:** Domain enrichment analysis for Ama-KTT/M/2 on α-amanitin versus Ama-KTT/M2 on no toxin (group 3 versus 2).

Domain	DEGs w/Domain	DEGs	Genes w/Domain	Genes	p-Value
Major royal jelly	4	2642	4	11890	0
Pupal cuticle protein C1	3	2642	3	11890	0
Cytochrome P450	51	2642	91	11890	1.20E-12
Cytochrome P450, conserved site	47	2642	82	11890	2.90E-12
Insect cuticle protein	55	2642	107	11890	1.91E-11
Chitin binding domain	51	2642	97	11890	3.38E-11
Cytochrome P450, E-class, group I	44	2642	81	11890	1.77E-10
Peptidase M17, leucyl aminopeptidase, N-terminal	8	2642	9	11890	4.38E-06
UDP-glucuronosyl/UDP-glucosyltransferase	20	2642	35	11890	4.93E-06
Leucine aminopeptidase/peptidase B	9	2642	11	11890	8.48E-06
Peptidase M17, leucyl aminopeptidase, C-term.	9	2642	11	11890	8.49E-06

This table shows the selected and significantly enriched domains in response to toxin treatment. “DEGs w/domain” are the differentially expressed genes that have a particular domain. “DEGs” is the number of all differentially expressed genes in this comparison. “Genes w/domain” is the total number of genes with a particular domain in the genome. “Genes” is the total number of genes in the genome. All p-values are corrected.

### The RT-qPCR Results Confirm the Microarray Data

**Figure 6:**
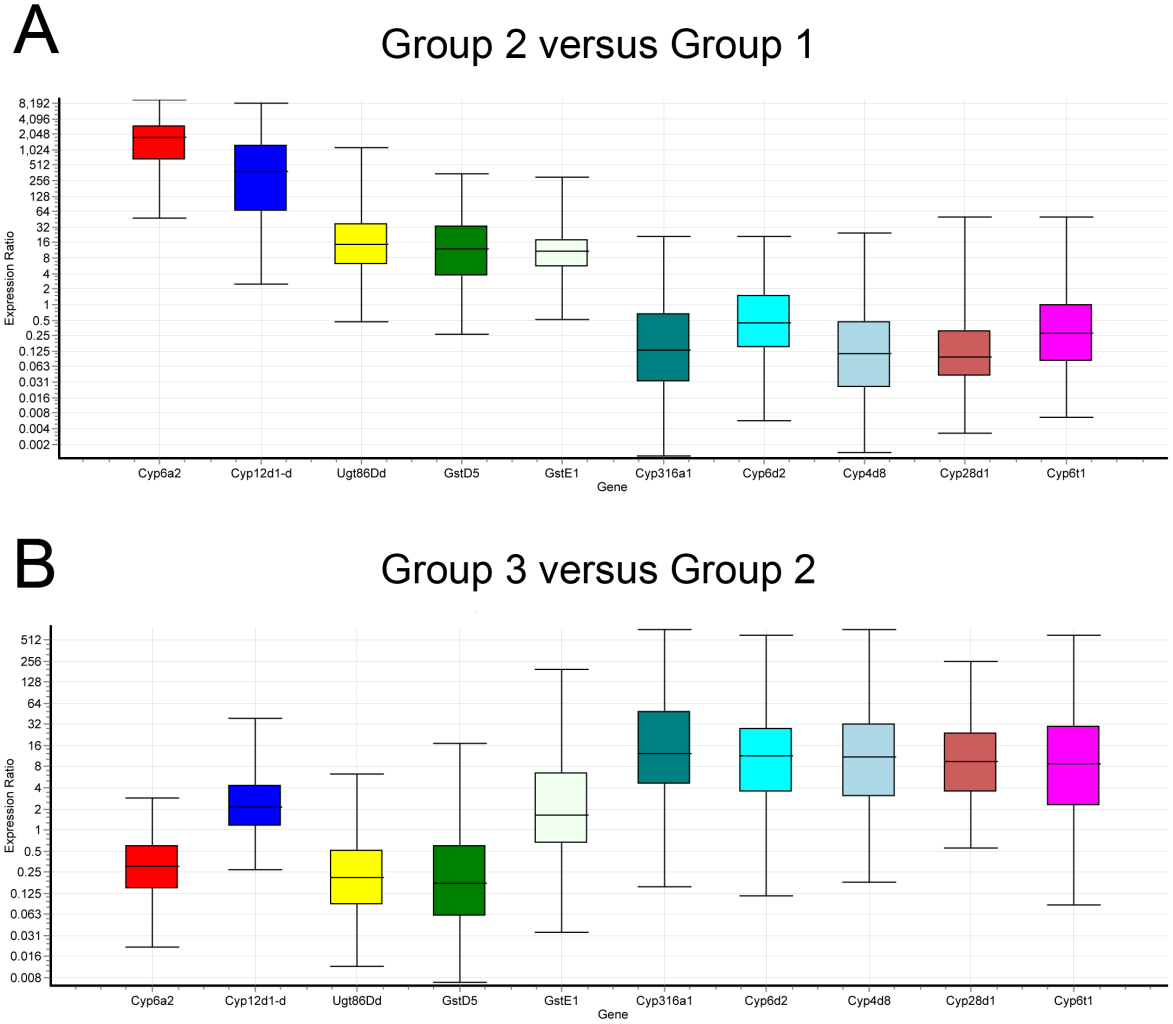
The qPCR results confirm the microarray data. A) Relative expression distribution (Y-axis) of ten selected genes is shown as a ratio comparing Ama-KTT/M/2 and Canton-S (group 2 versus group 1). Each measurement contains 15 replicates (3 replicates for each of the five biological controls of groups 1 and 2). B) Gene expression differences between Ama-KTT/M/2 treated with α-amanitin and Ama-KTT/M/2 (group 3 versus group 2) are compared. Group 3 contributes to 18 data points (three replicates for each of the six biological controls), while group 2 contributes to 15 data points, as previously mentioned. All comparisons were normalized with two reference genes, *Sucb* and *alpha-Tub84B*. Ratios above one indicate that a gene is up-regulated in the comparison.

We used real-time quantitative reverse transcription PCR (RT-qPCR) to confirm the fold-changes of ten genes, which we selected because of their high fold-changes and predicted importance for the resistance phenotype (Figure 6 and Table S5). When comparing Ama-KTT/M/2 to Canton-S (group 2 versus group 1), the genes *Cyp6a2*, *12d1-d*, *Ugt86Dd*, *GstD5*, and *GstE1* were between 1366.9 and 10.7-fold up-regulated (p<0.001 for all values, randomization test, B = 2000). When we compared Ama-KTT/M/2 treated with α-amanitin to Ama-KTT/M/2 (group 3 versus group 2), *Cyp316a1*, *6d2*, *4d8*, *28d1*, and *6t1* were up-regulated between 14.1 and 8.4-fold (p = 0.002 for *Cyp316a1* and p<0.001 for the other genes, randomization test, B = 2000). In summary, the microarray analysis fold-induction changes perfectly correlate with our RT-qPCR results, such that the microarray results slightly underestimate the fold-changes that resulted from the RT-qPCR analysis.

## Discussion

### Several Mechanisms Seem to Confer α-Amanitin Resistance

**Figure 7:**
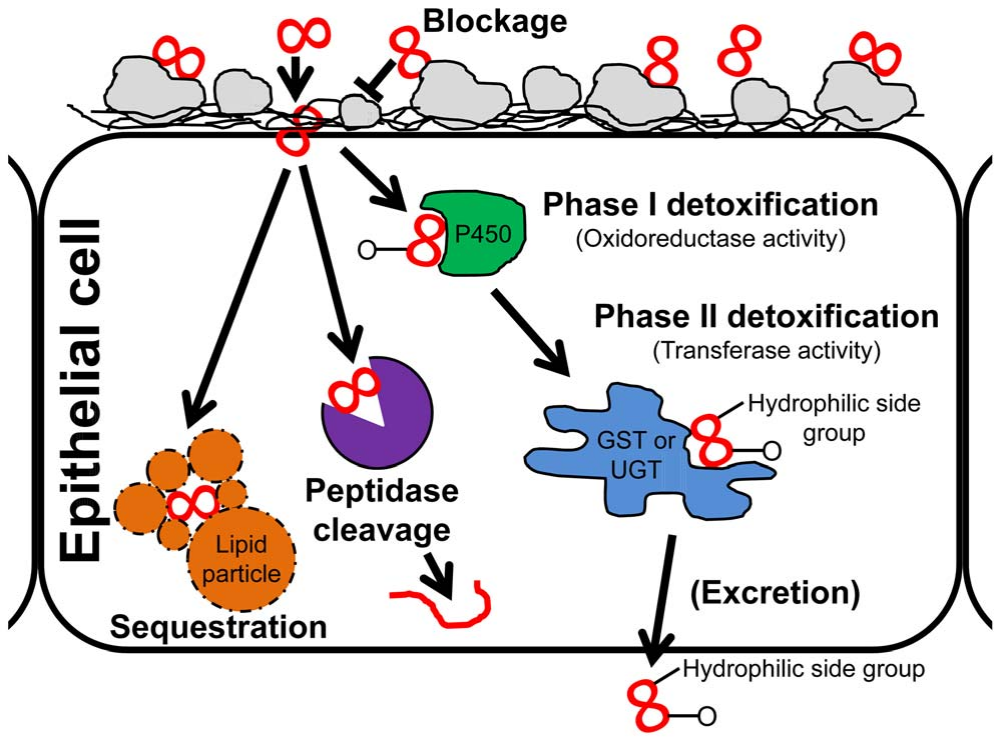
A model of the four mechanisms that contribute to the resistance to α-amanitin in concert. The bicyclic octapeptide α-amanitin is shown as a red 8. Cuticular proteins block some of the α-amanitin from entering the cells (blockage). α-Amanitin that entered the cytoplasm is either sequestered in lipid particles, cleaved by peptidases, or detoxified by phase I and II detoxification enzymes, possibly followed by excretion.

α-Amanitin is the principal toxin in some of the most deadly poisonous mushrooms, which inhibits the function of RNA-polymerase II by binding to it. Our results presented here comprise the first whole-transcriptome scale investigation to identify the molecular and cellular mechanisms that underlie the resistance to this very potent toxin in any organism. Using larvae of the resistant stock Ama-KTT/M/2 and the sensitive stock Canton-S, we identified both constitutive and α-amanitin-inducible mechanisms that can explain the resistance to α-amanitin in the Ama-KTT/M/2 stock. Based on an array of bioinformatics analyses of our microarray data and RT-qPCR validation, we found that four main mechanisms are likely to contribute in concert to the resistance: 1) constitutive and α-amanitin-inducible toxin entry blockage, mediated by cuticular proteins, the MRJP domain of the Yellow protein family, and Sgs proteins, 2) constitutive and α-amanitin-inducible phase I and II detoxification, mediated by the Cytochrome P450, GST, and UGT enzyme families (likely followed by excretion), 3) α-amanitin-inducible lipid particle gene induction, possibly leading to the sequestration of α-amanitin in cytoplasmic lipid particles, and 4) α-amanitin-inducible peptidase genes, perhaps leading to the digestion of α-amanitin either inside or outside (e.g. gut lumen) of cells (Figure 7).

In honey bee venom glands, the Major Royal Jelly Protein 8 (MRJP8) was shown to be a part of the cuticular layer that forms the inner lining of the gland. It was suggested that MRJP8 protects the venom gland cells from the stored toxins [49]. The closest relatives to the *MRJP* genes in *Drosophila* are the proteins of the Yellow family. The *yellow* gene itself was together with numerous cuticular protein genes identified as significant in our single gene, gene ontology, and domain enrichment analyses. It is thus possible that Yellow, together with other cuticular proteins, block the entry of α-amanitin into cells protected by a cuticular layer, such as the larval epidermis and gut epithelium (Figure 7). In a similar manner, the products of the five strongly α-amanitin-inducible salivary gland secretion genes *Sgs1*, *Sgs3*, *Sgs5*, *Sgs7*, and *Sgs8* (each >300-fold induced) could perhaps bind to α-amanitin and reduce its uptake in the midgut. Another possibility is that α-amanitin is simply a stress factor that induces these and other genes. After all, α-amanitin blocks messenger RNA transcription in poisoned cells, which is certainly stressful for the organism.

Besides being involved in environmental stress responses, hormone metabolism, and other metabolic functions, some Cytochrome P450, GST, and UGT proteins catalyze detoxification reactions, which transform a broad variety of xenobiotic substances into less toxic molecules that can be more easily excreted from the body [53]–[55]. Cytochrome P450 proteins, which are encoded by *Cyp* genes, are known for their broad range of substrates that they chemically modify. Several *Cyp* genes have been associated with single or multiple toxin resistance in diverse insect species, such as *Cyp6g1* [5], [28], [56]–[58], *Cyp6g2* [32], *Cyp6a2* [18]–[25], *Cyp12a4* [41], and *Cyp12d1* [22], [23], [26]–[32]. Our single gene and gene ontology enrichment analyses identified three of these detoxification-implicated *Cyp* genes, which are more than about 200-fold constitutively up-regulated in Ama-KTT/M/2: *Cyp6a2*, *Cyp12d1-d*, and *Cyp12d1-p* (Table 1). It is thus possible that one or all three of these genes contribute to the resistance to α-amanitin. There is also evidence that *Cyp12d1* is inducible by environmental stress factors, such as heat, oxidative stress, and air pollutants [29]–[31]. Because *Cyp6a2*, *Cyp12d1-d*, and *Cyp12d1-p* are constitutively up-regulated in our double-controlled study, stress is not a likely cause for the up-regulation of these three genes.

Some GST and UGT proteins perform phase II detoxification reactions that make toxic molecules bulkier and more hydrophobic, preparing the toxins for their excretion from the body. Several of these genes have been linked to insecticide resistance [6], [36], [54], [55], [59]–[71], while others are involved in several types of stress responses [34], [35], [45], [54]. Our single gene analysis showed that several *Gst* and *Ugt* genes are constitutively up-regulated in Ama-KTT-M/2 and that both gene families are significantly enriched in our gene ontology enrichment analysis, while their specific domains were identified as significant in the protein domain enrichment analysis. It is thus likely that some of them help detoxifying α-amanitin by making it both bulkier to prevent it from binding to RNA-Polymerase II and more water-soluble to augment its secretion via the Malpighian tubules (Figure 7). It is, however, possible that the α-amanitin-induced genes simply respond to stress caused by the effects of the toxin.

In our gene ontology enrichment analysis, we identified two other interesting mechanisms, which are inducible in response to α-amanitin in the larval food: the possible sequestration of α-amanitin in lipid particles and the cleavage of α-amanitin by peptidases. A group of genes involved in the cellular component ‘lipid particle’ were on average more than 5200-times induced by α-amanitin in the larval food. Natural and artificial lipid particles have been shown to be involved in various detoxification processes in very diverse organisms such as yeast, insects, and humans [50], [51], [72]. We therefore speculate that cytoplasmic lipid particles aggregate around α-amanitin molecules and trap them, thereby preventing the toxin from entering the nucleus, where RNA-Polymerase II performs its function. Furthermore, a variety of peptidase genes were identified in our various data analyses, suggesting that α-amanitin is cleaved either in the gut lumen, in the cells, or perhaps even in the food, if the larvae secrete peptidases from their mouths (Figure 7).

### Implications

Our data does not support the previously held view that an MDR mechanism confers α-amanitin resistance in *D. melanogaster*. In 1982 and 2000, two studies based on QTL mapping suggested that α-amanitin-resistance in four wild-caught *D. melanogaster* stocks is conferred by two major loci on chromosome 3 [14], [15], the more recent of which pointed out *Mdr65* and *Pkc98E* as possible candidates. However, our single gene and genome enrichment analyses identified two α-amanitin-inducible *Cyp* genes, *Cyp316a1* and *Cyp4d8*, which are situated close to the *Mdr65* locus and *Hr96* close to the *Pkc98E* locus. Because Begun and Whitley used QTL mapping, not deletion mapping, the two *Cyp* and the *Hr96* genes could instead be the resistance-conferring genes. Taking all the observations from our study together, we conclude that α-amanitin resistance has evolved as a quantitative complex trait that is based on entry blockage, phase I and II detoxification followed by secretion, peptidase cleavage, and sequestration.

Cross-resistance to a broad variety of toxins could explain how some *Drosophila* species evolved into mushroom-feeding specialists that can use mushroom toxins to their own advantage. For example, various mycophagous *Drosophila* species are frequently infected with parasitic nematodes that render about 20% of the adult flies sterile [11], [73]. Feeding on poisonous mushrooms not only kills the nematode parasites, it also provides a unique food source that is not accessible to many animals. *D. melanogaster* is a non-mycophagous species and should thus not be exposed to α-amanitin in nature. However, as discussed earlier, Cytochrome P450 enzymes can provide cross-resistance to multiple toxins, such as manufactured pesticides and natural xenobiotic products [32], [58]. We speculate that α-amanitin resistance in *D. melanogaster* has evolved in response to agricultural pesticides or other environmental factors, to which the flies were exposed before they were collected in the 1960s. Thus, if unrelated toxins can induce α-amanitin resistance, such a cross-resistance could prime a species to a radical host switch. If *D. melanogaster* females were to change their egg-laying behavior and oviposit on less toxic mushrooms, a niche change could result, followed by selection to feed on more toxic mushrooms. Being a species with such high fecundity, *D. melanogaster* could then even drive rare mycophagous *Drosophila* species out of their niche.

### Limitations

The most obvious limitation of every microarray is that the observations and conclusions are entirely based on mRNA transcription differences. It is thus possible that some important mechanisms escaped detection. Furthermore, many *D. melanogaster* genes are still poorly annotated and their true functions are elusive. We thus excluded the most poorly annotated genes from our analysis. However, in doing so, we might have inadvertently lost some important genes that could contribute to the resistance to α-amanitin. Furthermore, because we used whole larvae in our study, we cannot determine the relative importance that the different tissues play in the resistance to α-amanitin.

Our microarray data analysis did not reveal any gene-regulatory pathways that lead to the resistance to α-amanitin. *Hr96* and *cnc* have been shown to be upstream of detoxification genes [46], [47], [74]. *Hr96* is situated on the right arm of the third chromosome, where the genome enrichment analysis shows a peak in response to α-amanitin. However, the expression levels of both *Hr96* and *cnc* revealed nothing that would lead us to conclude their role in α-amanitin resistance. One reason for this could be that these genes encode transcription factors, which are already present in the cytoplasm to await activation, and we might not expect dramatic differences in their RNA regulation. Another reason could be that our larvae were feeding on α-amanitin from the first instar until they were collected at the late third instar. Thus, we might have missed the critical time period during which the upstream components of the pathway were up-regulated. We also noticed a lack of dramatic *Cyp*, *Gst*, and *Ugt* gene inducibility in response to α-amanitin. In the resistant stock Ama-KTT/M/2, many *Cyp*, *Gst*, and *Ugt* genes were constitutively expressed at higher levels than in Canton-S, while in larvae that were fed on toxic food, a completely different set of *Cyp* and *Gst* genes showed a much weaker induction than we initially expected. This weak gene induction is perhaps not surprising because in a previous microarray study using six different toxins, the detoxification gene families were not much inducible either [75]. It is thus possible that at least for the *Cyp*, *Gst*, and *Ugt* genes, the resistance to α-amanitin is mostly a constitutive trait.

Based on the mapping data from the two previous studies, we expected to find the α-amanitin resistance-conferring genes on chromosome 3 [14], [15]. Because the original Ama-KTT stock is 45 years old, we wanted to make sure that the genes on both major autosomes are homozygous before performing the microarray. One limitation to our approach is that we did not balance the X chromosome when we created the isochromosome stock Ama-KTT/M/2. However, we showed that the Ama-KTT/M/2 stock is not less resistant than original Ama-KTT stock (Figure 1), indicating that most if not all resistance-conferring alleles are present in the isochromosome stock that we used for the microarray. Most genes that we identified as significant are situated on chromosomes 2 and 3 (Tables 1 and 2). However, a few highly expressed genes, like *yellow*, are on the X chromosome. Thus, these X-chromosomal genes could either be the original alleles from Ama-KTT or the alleles from the multi-balancer stock. If they derived from the multi-balancer stock, the regulation of these genes could be explained by epistasis, such that the inducers of the X-chromosomal genes are situated on the two major autosomes, which are derived from the original Ama-KTT stock.

### Future Studies

In order to identify the upstream components of the pathways that lead to the resistance to α-amanitin in the Ama-KTT/M/2 isochromosome stock, future microarray studies should include samples of larvae that have been exposed to α-amanitin for different periods of time. Because first instar larvae are very small, the exposure to α-amanitin should happen during the third larval instar, and samples should be collected at a series of subsequent time points thereafter. This approach should be efficient to detect gene-regulatory differences of the upstream pathway components. Furthermore, it would be interesting to investigate the mechanisms that confer α-amanitin resistance in mycophagous *Drosophila* species, using the RNA sequencing approach. Mycophagous species are several orders of magnitude more resistant to α-amanitin than *D. melanogaster* [11]–[13]. The higher toxin resistance of those species could produce clearer signals for the determination of the factors that make *Drosophila* resistant. After we gain a clearer picture about the candidate genes that might confer α-amanitin resistance in several *Drosophila* species, the next step would be to provide conclusive genetic evidence if the candidate genes are sufficient and necessary for the α-amanitin resistance phenotype. This could be done by transgenically overexpressing the resistance-conferring alleles in either *D. melanogaster* or other sensitive species that are closely related to highly resistant mycophagous species. In *D. melanogaster*, overexpression of candidate genes can be achieved using the Gal4-UAS system with visible effects in different organs such as the gut, fat body, and Malpighian tubules [32], [58]. Such tests can reveal the organs and tissues that contribute to the resistance to α-amanitin. Because toxic mushrooms contain more than one toxin, mycophagous *Drosophila* species must be resistant to a variety of toxins that target different biological processes [12], [76], [77]. Thus, other commercially available mushroom toxins, such as β-amanitin, phalloidin, ibotenic acid, and muscimol should be used to test if cross-resistance or independent mechanisms provide protection against the variety of mushroom toxins that mycophagous larvae encounter in their food source. Another pressing question is where α-amanitin goes once it entered a larva. Is it digested in the gut? Does it enter the cytoplasm of all or just a subset of cells? Radioactive α-amanitin could be a means to answer this question, but the analysis of the data might prove very difficult.

## Conclusions

We suggest that the α-amanitin resistance phenotype in *D. melanogaster*, a species that does not feed on mushrooms in nature, has evolved as cross-resistance to pesticides or other factors in the environment. Entry blockage of α-amanitin into epithelial cells, phase I and II detoxification mediated by Cytochrome P450, GST, and UGT enzymes (likely to be followed by excretion from the body), sequestration of α-amanitin in cytoplasmic lipid particles, and proteolytic cleavage by peptidases are four likely mechanisms to contribute to the resistance phenotype in concert. In contrast, we did not detect any evidence for multidrug resistance efflux systems to be important for the resistance to α-amanitin. Future studies should include a time series of α-amanitin exposure, *Drosophila* species that actually feed on toxic mushrooms in nature, and more mushroom toxins. Candidate genes resulting from these experiments should then undergo sufficiency and necessity tests by transgenic rescue.

## Materials and Methods

### Fly Stocks

All fly stocks were maintained at room temperature on food containing Brewer's yeast, cornmeal, granulated sugar, agar, and methylparaben as antifungal agent. The wild-type stock Canton-S and the multi-balancer stock w[1118]/Dp(1;Y)y[+]; CyO/nub[1] b[1] sna[Sco] lt[1] stw[3]; MKRS/TM6B, Tb[1] were obtained from the Bloomington Stock Center, Bloomington, Indiana (stocks #1 and #3703, respectively). The α-amanitin-resistant Ama-KTT stock (# 14021-0231.07) was originally collected in 1968 in Kenting (Taiwan) and obtained from the Drosophila Species Stock Center at the University of California, San Diego.

### Generation of the Isochromosome Stock Ama-KTT/M/2

Because Ama-KTT was maintained in the absence of selective pressure to toxins in the stock center over the past five decades, the stock could have lost, or become heterozygous for, some of the α-amanitin resistance-causing alleles. In order to create flies homozygous for the resistance-conferring alleles, we crossed the Ama-KTT stock to the multi-balancer stock w[1118]/Dp(1;Y)y[+]; CyO/nub[1] b[1] sna[Sco] lt[1] stw[3]; MKRS/TM6B, Tb[1]. As a result, we created the isochromosome stock Ama-KTT/M/2, which is isogenic for the second and third chromosomes.

### Dose-Response Studies of the Fly Stocks to α-Amanitin

In order to quantify and compare the levels of α-amanitin resistance of the *D. melanogaster* stocks, dose-response experiments were performed, which measured the survival from freshly-hatched first-instar larvae to adulthood. Flies able to completely hatch from their pupae were scored as survivors. The α-amanitin-resistant stocks Ama-KTT and Ama-KTT/M/2 were tested on 11 α-amanitin concentrations, using 0 to 10 μg of α-amanitin per g of food in 1 μg increments. The α-amanitin-sensitive wild-type stocks Canton-S and the multi-balancer stock w[1118]/Dp(1;Y)y[+]; CyO/nub[1] b[1] sna[Sco] lt[1] stw[3]; MKRS/TM6B, Tb[1] were initially tested on five concentrations ranging from 0 to 4 μg of α-amanitin per g of food in 1 μg increments. However, because they survived only the zero-concentration, these stocks were further tested on 0, 0.25, 0.5, 0.75, 0.1, 0.25, and 0.375 μg of α-amanitin per g of food.

In order to obtain first-instar larvae for the dose-response experiments, flies of mixed sexes were allowed to lay eggs on molasses agar caps that contained a streak of fresh Baker's yeast paste at 25°C, 70% humidity, and a 12∶12 hour day/night cycle. The yeast was removed prior to larval hatching. Freshly hatched first-instar larvae were placed in groups of ten into 2-mL plastic test tubes (USA Scientific), each containing 500 mg of non-toxic or poisoned food and two small air holes in the lid. The food consisted of 125 mg dry, instant *Drosophila* medium (Carolina) and 375 μL sterile Milli-Q water with or without dissolved α-amanitin. Ten tubes were prepared for each toxin concentration and experimental replicate, resulting in 100 larvae for each concentration and experiment. Three high-quality dose-response experiments, in which the zero-concentration survival rate was at least 80%, were used to calculate the LC_50_ of each fly stock. The standard deviation of the mean (s.e.m.) was calculated for each concentration by sampling the data points of all 30 vials of every concentration. The LC_50_ was calculated using scatter plots and the logarithmic trendline function in Microsoft Excel.

### Sample Preparation for the Microarray Analysis

In order to compare the constitutive gene-regulatory differences across the entire transcriptome between α-amanitin-sensitive and -resistant stocks, freshly-hatched first-instar larvae of the sensitive Canton-S stock (group 1) and the resistant Ama-KTT/M/2 stock (group 2) were placed in groups of ten into 2-mL plastic test tubes (USA Scientific), containing 500 mg of non-toxic food. To identify the genes that are inducible by α-amanitin, Ama-KTT/M/2 larvae were raised on 1.5 μg of α-amanitin per g of food (group 3), which is slightly lower than the LC_50_ concentration of this stock. All larvae were raised until they reached the late third instar at 25°C, 70% humidity, and a 12∶12 hour day/night cycle. Because not all larvae survived in the tubes and the larvae on α-amanitin-containing food had a slower growth rate, initially 600 first-instar larvae (60 tubes) for each group were started over three subsequent days (20 tubes per group and day). When the majority of larvae reached the late third instar, the tubes were emptied and groups of ten late, but still feeding third-instar larvae were randomly picked from across all tubes and flash-frozen in batches of ten in liquid nitrogen, each batch providing the RNA for one microarray chip. Five biological replicates (ten larvae each) were prepared for groups 1 and 2, whereas group 3 was prepared in six biological replicates (ten larvae each). All samples were collected on the same morning. RNA extraction was performed without delay, using the RNeasy microarray tissue kit (Qiagen), according to the manufacturer's instructions.

### Affymetrix Array Target Preparation, Hybridization, and Scanning

Collection and analysis of data were compliant with MIAME standards [78]. The microarray experiment was performed using the Affymetrix GeneChip Drosophila Genome 2.0 Arrays (Affymetrix, Santa Clara, CA, USA) with biotinylated targets derived from total RNA. Each array contains 18,952 probes that interrogate ∼18500 transcripts of genes present in the transcriptome of *D. melanogaster*. Prior to labeling, total RNA samples were checked for purity and concentration, using a NanoDrop ND-1000 spectrophotometer (Thermo Fisher, Waltham, MA, USA) and for integrity, using RNA 6000 Nano Chips in a BioAnalyzer 2100 (Agilent Technologies, Santa Clara, CA, USA). cDNA for hybridization was synthesized and biotin-labeled from 400 ng of total RNA, using a MessageAmp Premier IVT kit (Ambion, Austin, TX, USA) according to the manufacturer's specifications. Biotinylated cDNA was fragmented, then hybridized, washed, and stained using a GeneChip Hybridization, Wash, and Stain Kit (Affymetrix, Santa Clara, CA, USA) according to the manufacturer's specifications. Arrays were post-processed on the AFX 450 Fluidics Station before they were scanned on an AFX GC3000 G7 Scanner (Affymetrix, Austin, TX, USA). Data were extracted from the raw images, using the Affymetrix Expression Console v.1.2 software. The RNA quality check, labeling, hybridization, and imaging procedures were performed according to Affymetrix protocols at the Center for Genomics Research and Biocomputing, University of Wisconsin.

### Microarray Data Normalization

The quality of microarray data sets was first checked by examining the distribution of the Studentized deleted residuals, using a previously described procedure [79], [80], and only high-quality microarray data were used for normalization. Probeset-level normalization was performed with the PLIER (Probe Logarithmic Intensity Error) algorithm with quantile normalization and mismatch intensity adjustment, using the Affymetrix Power Tools software v.1.14.4.1. Probesets were annotated using release 32 of the Affymetrix annotation for the Drosophila 2.0 array platform. The CEL files and summarized (normalized) microarray data resulting from this study have been deposited in the NCBI's Gene Expression Omnibus database at NIH (http://www.ncbi.nlm.nih.gov/geo/) with the accession number of GSE52782.

### Genome Enrichment Analysis

To find genome regions containing more differentially expressed genes than expected by chance, we used the binomial coincidence detection algorithm [81] with modifications specific for this dataset. Because *D. melanogaster* has a smaller genome and shorter regions of genetic linkage than mammalian genomes, we reduced the length of the overlapping bins to 500 kb spaced at 250 kb intervals. In order to reduce the total noise and find the strongest signal, we used only the top 0.01 most differentially expressed genes in the dataset. Briefly, under a null hypothesis of no significant enrichment in a genome region, the probability of finding a significantly differentially expressed gene within each bin will follow a binomial distribution with a probability of any given gene being significantly differentially expressed at no more than 0.01. The algorithm calculates a binomial probability for the empirical quantity of differentially expressed genes within each bin across the entire genome. The decimal log of the inverse of these probabilities is graphed. A decimal log of 2 corresponding to the horizontal line through each graph indicates a probability of a cluster occurring 1 in 100 times under the null hypothesis, the cutoff used for this method. The resulting graphic shows clustering over the whole genome and spikes indicate clusters unlikely to have occurred by chance. This is statistical evidence that a genome region is likely implicated in a gene expression phenotype. The assumptions for the inferential statistics used for this analysis necessitate inclusion of low copy genes as differentially expressed, thus the inferential statistics used to generate the genome enrichment figure were performed in the limma package in Bioconductor v.2.10 [82]. Cytoband visualization is derived from annotation tables of the UCSC dm3 genome, which represents cytobands as alternating light and dark bands.

### Identification of Differentially Expressed Genes (DEGs)

A nonparametric method, RankProd (RP) [83], was used to identify differentially expressed genes (DEGs) between Ama-KTT treated with α-amanitin slightly below the LC_50_ concentration, and untreated Ama-KTT, or Canton-S conditions. We chose RP because it had been implicated to be more accurate for ranking genes by differential expression than t-statistics or derived methods [84]. Kadota *et al.* once evaluated eight DEG ranking methods and concluded that RP is one of the best performing methods [84]. Laing *et al.* indicated RP is one of most efficient method when replicate numbers is less than 10 [85]. In this study, we applied multiple testing corrections to the p-values resulting from RP using Benjamini and Hochberg False Discovery Rate [86] and all genes with corrected p-values (<0.05) were defined as DEGs.

### Gene Ontology Enrichment Analysis

The DEGs identified from each comparison, namely, Ama-KTT/M/2 versus Canton-S and Ama-KTT/M/2 on α-amanitin versus Ama-KTT/M/2 on no toxin, were used as the input for the gene ontology enrichment analysis. We employed an online tool, AmiGO's Term Enrichment, to identify the enriched gene ontologies (http://amigo.geneontology.org/). This tool uses the Perl module GO:TermFinder available at CPAN (http://search.cpan.org/) to identify the enriched gene ontology terms associated with a DEG list, using the hypergeometric probability function. We applied multiple testing corrections to calculate the p-values of all GO terms and then corrected p-values using Benjamini and Hochberg False Discovery Rate [86]. All gene ontology terms with a corrected p-value <0.05 were considered to be significantly enriched.

### Protein Domain Enrichment (PDE) Analysis

Protein domains were analyzed with InterproScan [87]. We first downloaded and installed InterproScan and associated databases to our Linux server and performed the standalone analysis to identify protein domains of all target sequences provided by FlyBase (http://flybase.org/static_pages/docs/datafiles.html). The enrichment of each domain in the differentially expressed gene list was compared to the occurrence of the respective domain in the background of all genomic genes, and two parameters were introduced to show the enrichment of each domain as described in [88]: (1) Enrichment factor, EF = k/(nM/N); and (2) the E_score, which is the hypergeometric probability of identifying at least k domains from DEG list. It is calculated using the following formula: 
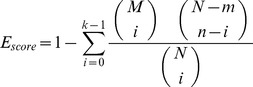



N is the total number of domains associated with all genomic genes, M is total number of a specific domain for all genes in the genome, n is the number of all domains associated with the DEGs, and k is the number of a specific domain present in the DEGs list. We applied multiple testing corrections to the p-values calculated via hypergeometric probability using Benjamini and Hochberg False Discovery Rate (FDR) [86]. The significantly enriched protein domains are those that have a corrected p-value <0.05.

### RT-qPCR Validation of the Microarray Results

Quantitative real-time PCR (qPCR) was performed on ten genes of interest to confirm the results of the microarray analysis. Each gene of interest and biological replicate was repeated three times to ensure the statistical significance of the result. The genes included two *Cyp*, one *Ugt*, and two *Gst* genes that were up-regulated when comparing the resistant group Ama-KTT/M/2 to the control group Canton-S (group 2 versus group 1) and five *Cyp* genes that were up-regulated when comparing Ama-KTT/M/2 on α-amanitin to Ama-KTT/M/2 on no toxin (group 3 versus group 2). Two reference genes, *Scub* and *alpha-tub84B*, were used as controls to normalize the results. These genes were selected because their fold-changes were nearly zero for each comparison. The primer pairs used were a part of the Taqman Gene Expression Assays kit (Applied Biosystems): Dm02361072_s1, Dm01831596_g1, Dm01840671_g1, Dm01830394_g1, Dm01822311_g1, Dm01804633_g1, Dm01799869_s1, Dm02147253_g1, Dm01817955_g1, Dm02152265_s1, Dm01826948_s1, and Dm02374415_g1. The reactions were performed in a StepOnePlus Real-Time PCR System (Applied Biosystems). The High Capacity cDNA Reverse Transcription Kit (Applied Biosystems) was used to reverse transcribe RNA to cDNA in an Eppendorf PCR machine for 96 reactions (Eppendorf, Model 96S). We used REST 2009 to calculate the RT-qPCR p-values.

## Supporting Information

Table S1 Differentially expressed genes (DEGs) between Ama-KTT/M/2 on no toxin versus Canton-S (group 2 versus 1), Ama-KTT/M/2 on toxin versus Canton-S (group 3 versus 1), and Ama-KTT/M/2 on toxin versus Ama-KTT/M/2 on no toxin (group 3 versus 2). This table contains 4209 DEGs that are differentially expressed in at least one of the three comparisons.
(XLSX)

Table S2 Complete single gene analysis for Ama-KTT/M/2 versus Canton-S on no toxin (group 2 versus 1). This table contains well-annotated genes that are at least 2.0-fold constitutively up-regulated in the resistant stock, as compared to the sensitive stock, on no toxin. The p-value cutoff is p<0.05.
(XLSX)

Table S3 Complete single gene analysis for Ama-KTT/M/2 on toxin versus Ama-KTT/M/2 on no toxin (group 3 versus 2). This table contains well-annotated genes that are at least 2.0-fold inducible by feeding larvae of the resistant stock with α-amanitin-containing food, as compared to resistant larvae on no toxin. The p-value cutoff is p<0.05.
(XLSX)

Table S4 Genome enrichment analysis for group 2 versus 1 and group 3 versus 2. This table shows the genes behind the peaks in Figure 3. The peak at band 38B is the only locus that is differentially expressed between Ama-KTT/M/2 and Canton-S on no toxin (group 2 versus group 1). The remaining peaks 66A, 69A, 92A, and 96D show differentially expressed loci in response to α-amanitin treatment (group 3 versus group 2). All p-values are corrected and fold-changes are given for the individual genes. Peaks 66A and 96D are very close to the two QTL mapping peaks identified in previous studies [14], [15].
(XLSX)

Table S5 Comparison of qPCR and microarray fold-induction values. The first five genes were constitutively over-expressed in Ama-KTT/M/2, as Compared to Canton-S (Group 2 versus group 1). The last five genes were induced by α-amanitin in Ama-KTT/M/2, as compared to Ama-KTT/M/2 on no toxin (group 3 versus group 2). The RT-qPCR p-values are uncorrected, while the array p-values are corrected.
(XLSX)
